# Real-world impact of mepolizumab on pediatric and adolescent patients with severe asthma

**DOI:** 10.1016/j.jacig.2025.100638

**Published:** 2025-12-20

**Authors:** Michelle L. Hernandez, Tom Corbridge, François Laliberté, Malena Mahendran, Annalise Hilts, Kaixin Zhang, Arijita Deb

**Affiliations:** a Division of Allergy and Immunology, Department of Pediatrics, University of North Carolina School of Medicine, Chapel Hill, NC; b Medical Affairs, GSK, Durham, NC; c Groupe d’analyse, Ltée, Montreal, Quebec, Canada; d Global Real-World Evidence and Health Outcomes Research, GSK, Upper Providence, Pa

**Keywords:** Mepolizumab, pediatric, adolescent, corticosteroid, severe asthma, exacerbation, eosinophilic phenotype, health care resource utilization, short-acting β_2_-agonist

## Abstract

**Background:**

Real-world evidence on the effectiveness of mepolizumab in children and adolescents with severe asthma (SA) is limited.

**Objective:**

We sought to evaluate mepolizumab’s impact on the clinical and health care resource utilization (HCRU) burden of SA in children and adolescents.

**Methods:**

A retrospective study (GSK ID: 218952) was conducted of US administrative claims for patients aged 6-17 years with SA who initiated mepolizumab (from October 1, 2016, to June 30, 2023), had continuous health plan enrollment for ≥12 months pre- and post-mepolizumab initiation, and had ≥1 additional mepolizumab dispensings/administrations ≤6 months from initiation. Rate ratios from Poisson regression models were used to compare asthma exacerbations, oral corticosteroid (OCS) dispensings and bursts, short-acting β_2_-agonist (SABA) canister use, and HCRU per patient-year (PPY) pre- and post-mepolizumab; risk ratios from log-binomial regression models compared proportions of patients with ≥1 OCS dispensings and ≥1 SABA canister dispensings between periods.

**Results:**

Of 580 patients, 47% were aged 6-11 years and 53% were aged 12-17 years. Mean OCS dispensings PPY decreased by 24% (*P* < .001) pre- versus post-mepolizumab initiation. Mean overall asthma exacerbations and OCS bursts PPY decreased by 34% and 29% (*P* < .001 each), respectively. The proportions of patients with ≥1 OCS dispensings and those using ≥1 SABA canisters decreased by 16% (*P* < .001) and 3% (*P* = .039), respectively. Asthma-related HCRU PPY decreased by 23% for inpatient visits (*P* = .031), 15% for emergency department visits (*P* = .021), and 26% for outpatient visits (*P* < .001).

**Conclusions:**

Mepolizumab initiation was associated with significant reductions in asthma exacerbations, OCS and SABA use, and HCRU in children and adolescents with SA, demonstrating real-world clinical benefit.

In the United States, approximately 10% of children aged 0-17 years are diagnosed with asthma.^1^ Although many of these children can be managed with low to medium inhaled corticosteroid (ICS) doses, up to 10% of child asthma cases are severe,^2^^,^^3^ for which the associated economic burden and health care resource utilization (HCRU) are substantial.^2^^,^^4^, ^5^, ^6^ In the United States, certain racial or ethnic groups are disproportionately impacted by asthma: severe and poorly controlled asthma disproportionately affects children from racially underserved populations, particularly those identified as Black or African American,^7^ and asthma-related mortality rates and emergency department (ED) visits are 2- and 6-fold higher, respectively, among Black individuals compared with White individuals.^8^ In this article, we use racial and ethnic categories as social constructs that reflect lived experiences and structural inequities, not inherent biological differences.

Severe asthma (SA) is defined as asthma that remains uncontrolled despite adherence to optimized high-dose ICS with a long-acting β_2_-agonist or that worsens when such treatment is stepped down.^9^ Add-on treatments for SA in children and adolescents include long-acting muscarinic antagonists, leukotriene receptor antagonists, and biologics that target a specific inflammatory phenotype (eg, anti-IgE, anti-IL-4 receptor ⍺, anti–IL-5, or anti–thymic stromal lymphopoietin therapies).^9^ During poor asthma control periods, short-acting β_2_-agonists (SABAs) are commonly used to relieve acute symptoms, despite leaving the underlying airway inflammation untreated.^10^ Short courses (or bursts) of oral corticosteroids (OCSs) can also be used, but are generally considered a last resort (for patients aged ≥6 years) due to the substantial and cumulative side effects of steroid usage and the increasing availability of biologic therapies as an alternative.^9^^,^^11^ In children, long-term daily use of high-dose ICSs may be associated with diminished linear growth^12^ and adrenal suppression;^13^ high SABA use is associated with increased risk of asthma-related hospitalizations, exacerbations, need for OCS bursts, and asthma-related deaths.^14^^,^^15^ Thus, a key management goal in the treatment of pediatric patients with SA is to initiate add-on therapy that controls the disease and is well tolerated, reducing the need for OCSs and SABAs. This has driven the use of biologics for the treatment of SA in children aged ≥6 years, which now includes omalizumab, mepolizumab, dupilumab, and benralizumab,^16^, ^17^, ^18^, ^19^ with tezepelumab approved for adolescents aged 12-17 years.^20^

Mepolizumab is an anti-IL-5 mAb, approved as add-on maintenance treatment for patients aged ≥6 years with SA with an eosinophilic phenotype.^18^ IL-5 is a cytokine that is central to type 2 inflammation, and inhibition of IL-5 by mepolizumab has been shown to reduce eosinophilic inflammation in addition to having direct and indirect effects on various structural and inflammatory cells.^21^^,^^22^ Reductions in asthma exacerbations and OCS and SABA use in children, adolescents, and adults with SA have been demonstrated in clinical trials and real-world studies of mepolizumab, although the numbers of enrolled patients <18 years of age was low.^22^, ^23^, ^24^, ^25^, ^26^, ^27^, ^28^, ^29^ A phase 2 clinical trial in children aged 6-11 years demonstrated a decline in exacerbation rates and related hospitalizations with mepolizumab,^30^ and another recent phase 2 randomized trial of patients aged 6-17 years living in urban neighborhoods (Mechanisms Underlying Asthma Exacerbations Prevented and Persistent with Immune Based Therapy: A Systems Approach Phase 2 [MUPPITS-2]) found that mepolizumab decreased the annualized rate of asthma exacerbations requiring systemic corticosteroids (SCSs) compared with placebo.^31^

Real-world evidence on the association of mepolizumab initiation with clinical outcomes in pediatric and adolescent populations remains sparse. Therefore, this study aimed to provide a comprehensive assessment of the effects of mepolizumab on the clinical and HCRU burden of SA in patients aged 6-17 years.

## Methods

### Study design and data source

This was a retrospective, self-controlling, observational study with a pre- and post-mepolizumab cohort, using US administrative claims data from the Komodo Research Database (GSK ID: 218952). The Komodo Research Database compiles data from various payers (Medicaid, commercial, and Medicare insurance providers) and health care organizations. Pediatric and adolescent patients with SA who initiated mepolizumab treatment between October 1, 2016, and June 30, 2023, were assessed for eligibility (see Fig E1 in this article’s Online Repository at www.jaci-global.org). The index date was defined as the first dispensing/administration of mepolizumab on or after October 1, 2016; the pre-mepolizumab period was the 12 months leading up to and including the index date, and the post-mepolizumab period encompassed the 12 months following the index date.

### Patient population

Eligible patients were aged 6-17 years on the index date with ≥1 medical claims at any position with a diagnosis code for asthma (*International Classification of Diseases, Tenth Revision*: J45.3x-J45.5x and J45.9xx)^32^ during the pre-mepolizumab period or on the index date, and ≥1 medical or pharmacy claims for mepolizumab. SA was defined according to the Global Initiative for Asthma guidelines.^9^ Patients were also required to have ≥1 additional claims for mepolizumab ≤6 months from the index date and to have continuous enrollment in a plan with medical and pharmacy benefits for ≥12 months before and after the index date. Patients were not eligible if they had a medical or pharmacy claim for mepolizumab any time before the index date or a medical or pharmacy claim for omalizumab, reslizumab, benralizumab, dupilumab, or tezepelumab use during the study period.

### Outcomes

The primary end points were the rates of OCS dispensings and asthma exacerbations (including inpatient [IP]-, ED-, and SCS-defined exacerbations) compared between the pre- and post-mepolizumab initiation periods. Exacerbations defined by IP or ED visits involved hospitalization with a primary asthma diagnosis occurring ≤1 day after. An SCS-defined exacerbation was an ED or outpatient (OP) visit with a primary asthma diagnosis, accompanied by an OCS or SCS claim ≤5 days. If ≥2 exacerbations occurred within 14 days, they were considered a single episode.

Secondary end points included OCS treatment patterns compared between the pre- and post-mepolizumab initiation periods. These encompassed the rate of OCS bursts (pharmacy claim for OCS with 2-28 days of supply and a mean/median daily dose of ≥20 mg prednisone equivalent), the mean daily dose of OCS per period, and the percentages of patients with ≥1 OCS dispensings, maintenance OCS use (≥1 pharmacy claim for an OCS dispensing with >28 days of supply), and chronic OCS use (mean daily dose of ≥5 mg or ≥10 mg prednisone equivalent per period or ≥10 mg in the last 90 days). Additional secondary end points quantified SABA use (rate of SABA canister use and percentage of patients using ≥1 SABA canister), ICS dose (proportion of patients with ≥1 dispensing for low-dose, medium-dose, and high-dose ICSs), and all-cause, asthma-related, and asthma exacerbation-related HCRU based on IP, ED, and OP visits. Other visits (related to durable medical equipment or dental or vision services) were assessed for all-cause and asthma-related HCRU only.

### Statistical analysis

Patient demographic characteristics on the index date and clinical characteristics during the pre-mepolizumab period were described. Rates of asthma exacerbations, OCS dispensings, OCS bursts, SABA canister use, and HCRU were evaluated per patient-year (PPY) and compared between the pre- and post-mepolizumab periods using rate ratios estimated from Poisson regression models with generalized estimating equations (GEEs). The percentages of patients with ≥1 OCS dispensings, maintenance OCS use, chronic OCS use, ≥1 SABA canister dispensings, and ≥1 dispensings for low-, medium-, and high-dose ICSs were compared between these periods using risk ratios estimated from log-binomial regression models with GEEs. The mean daily OCS dose for each period was compared using mean differences estimated from linear regression models with GEEs. Each regression model provided 95% CIs and *P* values. These analyses were also stratified by age group: pediatric patients (6-11 years) and adolescent patients (12-17 years). Finally, a sensitivity analysis was performed to address the potential confounding effect of the coronavirus disease 2019 (COVID-19) pandemic. The rates of asthma exacerbations, OCS and SABA treatment patterns, and HCRU were compared before and after mepolizumab initiation among adolescent patients (aged 12-17 years) in the period before the COVID-19 pandemic (October 1, 2016, to March 1, 2020). Accordingly, the required mepolizumab initiation period was shortened (October 1, 2016, to March 1, 2019) for the sensitivity analysis.

### Ethics

No direct patient contact or primary collection of individual patient data occurred. Study results and aggregate analyses omitted patient identification, and therefore informed consent or ethics committee/institutional review board approval were not required. De-identified data were used and the data complied with the requirements of the Health Insurance Portability and Accountability Act.

## Results

### Patient population

**Fig 1:**
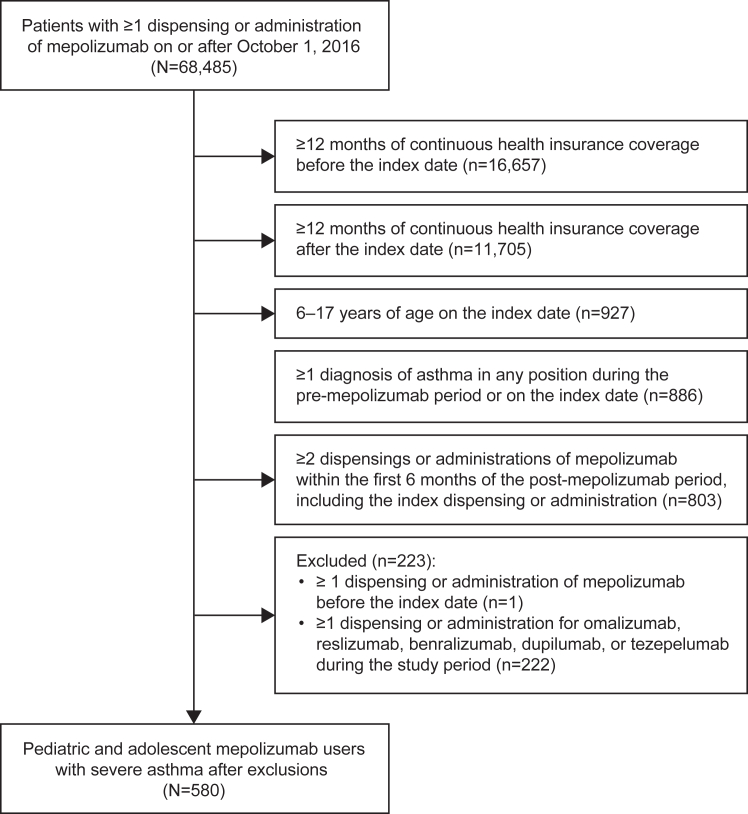
Flowchart of patient selection process and patient disposition.

Overall, 275 pediatric and 305 adolescent patients (N = 580) with SA were included in this study (Fig 1). Of these, 64.5% were male, 33.1% were Black, 24.7% were White, and 16.7% had Hispanic ethnicity (Table I). Mean ± standard deviation age at the index date was 11.9 ± 2.8 years. Most patients (76.9%) were enrolled in Medicaid, with the remainder covered by commercial health insurance. The most common asthma-related comorbidities in the 12 months before the index date were allergic rhinitis (86.0%), upper respiratory tract infection (61.6%), and sinusitis (25.7%). During the same period, nearly all patients used maintenance controller medications (95.9%) and rescue medications (97.8%). The most commonly used maintenance controller medications were ICSs (93.6%) and ICS/long-acting β_2_-agonist combination (88.4%), and rescue medications included SABAs (93.4%) and SCSs (85.3%).

**Table I:** Patient demographic characteristics on the index date and clinical characteristics during the pre-mepolizumab period

Characteristics	Pediatric and adolescent patients with SA (N = 580)
Age (y), mean ± SD (median)	11.9 ± 2.8 (12)
Age category, n (%)	
6-11 y	275 (47.4)
12-17 y	305 (52.6)
Sex, female, n (%)	206 (35.5)
Region, n (%)	
South	315 (54.3)
Midwest	99 (17.1)
Northeast	95 (16.4)
West	71 (12.2)
Insurance plan type, n (%)	
Medicaid	446 (76.9)
Commercial	125 (21.6)
Race/ethnicity, n (%)	
Black or African American	192 (33.1)
White	143 (24.7)
Hispanic or Latino	97 (16.7)
Asian or Pacific Islander	8 (1.4)
Other/unknown	140 (24.1)
Quan-CCI, mean ± SD (median)	1.0 ± 0.3 (1)
Asthma-related comorbidities, n (%)	
Allergic rhinitis	499 (86.0)
Upper respiratory tract infection	357 (61.6)
Food allergy	166 (28.6)
Sinusitis	149 (25.7)
Obesity	111 (19.1)
Gastroesophageal reflux disease	107 (18.4)
Obstructive sleep apnea	72 (12.4)
Pneumonia	68 (11.7)
Anxiety disorders	67 (11.6)
Acute respiratory failure	60 (10.3)
Maintenance controller medications, n (%)	556 (95.9)
ICS	543 (93.6)
ICS/LABA	513 (88.4)
Leukotriene modifiers	447 (77.1)
LAMA	164 (28.3)
Maintenance OCS use, n (%)	64 (11.0)
Chronic OCS use, n (%)∗	43 (7.4)
Rescue medications, n (%)	567 (97.8)
SABA	542 (93.4)
SCS	495 (85.3)
Antibiotics	348 (60.0)
SAMA	147 (25.3)
SABA/SAMA	76 (13.1)
AMR, mean ± SD (median)	0.5 ± 0.2 (0)

*AMR*, Asthma medication ratio; *LABA*, long-acting β_2_ agonist; *LAMA*, long-acting muscarinic antagonist; *Quan-CCI*, Quan-Charlson Comorbidity Index; *SAMA*, short-acting muscarinic antagonist.

∗ Mean daily dose ≥ 5 mg.

### OCS dispensings and asthma exacerbations

**Fig 2:**
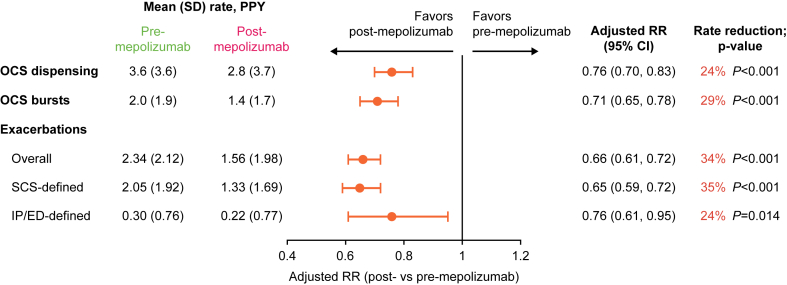
Rates of OCS dispensings, OCS bursts, and exacerbations pre- and post-mepolizumab. *RR*, Rate ratio.

The mean number of OCS dispensings PPY significantly decreased by 24% pre- versus post-mepolizumab initiation (*P* < .001), and the mean number of overall asthma exacerbations PPY significantly decreased by 34% (*P* < .001) (Fig 2). The reduction in the rate of exacerbations was driven by a 24% decrease in IP-/ED-defined exacerbations (*P* = .014) and a 35% decrease in SCS-defined exacerbations (*P* < .001). Significant reductions in the rates of OCS dispensings and overall asthma exacerbations PPY were also observed when patients were stratified by age into pediatric and adolescent groups (see Tables E1 and E2 in this article’s Online Repository at www.jaci-global.org).

### OCS treatment patterns, SABA use, ICS dosage, and HCRU

The mean number of OCS bursts PPY decreased from 2.0 to 1.4, representing a significant rate reduction of 29% pre- to post-mepolizumab initiation (*P* < .001) (Fig 2). The percentage of patients receiving ≥1 OCS dispensings also significantly decreased by 16%, from 82.6% pre-mepolizumab initiation to 69.7% post-mepolizumab initiation (*P* < .001) (Table II). Although the percentage of patients using maintenance OCSs decreased following mepolizumab initiation and fewer patients had chronic OCS use, these reductions were not statistically significant. The mean daily dose of OCSs per period significantly decreased from 1.9 mg to 1.5 mg pre- versus post-mepolizumab initiation (*P* < .001). Significant reductions in the rate of OCS bursts PPY, percentage of patients receiving ≥1 OCS dispensings, and mean daily dose of OCS per period were also observed after age stratification (Tables E1 and E2).

**Table II:** OCS treatment patterns, SABA use, and ICS dosage pre- vs post-mepolizumab

OCS, SABA and ICS use	Pre-mepolizumab period (N = 580)	Post-mepolizumab period (N = 580)	Measures of effect (95% CI)	*P* value
OCS treatment patterns			*Risk ratio*	
Patients with ≥1 OCS dispensing, n (%)	479 (82.6)	404 (69.7)	0.84 (0.80 to 0.89)	<.001
Maintenance OCS use, n (%)	64 (11.0)	54 (9.3)	0.84 (0.66 to 1.08)	.182
Chronic OCS use, n (%)				
Mean daily dose ≥5 mg per period	43 (7.4)	39 (6.7)	0.91 (0.68 to 1.22)	.517
Mean daily dose ≥10 mg per period	6 (1.0)	5 (0.9)	0.83 (0.32 to 2.15)	.706
Mean daily dose ≥10 mg in the last 90 d	25 (4.3)	15 (2.6)	0.60 (0.34 to 1.04)	.071
			*Mean difference*	
Daily OCS dose per period, mg, mean ± SD (median)	1.9 ± 2.1 (1.4)	1.5 ± 2.1 (0.8)	–0.40 (–0.54 to –0.27)	<.001
SABA use			*Rate ratio*	
No. of SABA canisters PPY, mean ± SD (median)	7.1 ± 5.9 (6.0)	7.0 ± 6.3 (5.0)	0.98 (0.93 to 1.04)	.553
			*Risk ratio*	
Patients with ≥1 SABA canisters, n (%)	530 (91.4)	514 (88.6)	0.97 (0.94 to 1.00)	.039
ICS dosage, n (%)			*Risk ratio*	
Patients with ≥1 dispensing for low-dose ICS	55 (9.5)	46 (7.9)	0.84 (0.63 to 1.12)	.226
Patients with ≥1 dispensing for medium-dose ICS	177 (30.5)	127 (21.9)	0.72 (0.62 to 0.83)	<.001
Patients with ≥1 dispensing for high-dose ICS	489 (84.3)	490 (84.5)	1.00 (0.97 to 1.04)	.906

The mean number of SABA canisters PPY was not significantly different pre- versus post-mepolizumab initiation (Table II). However, the percentage of patients using ≥1 SABA canisters significantly decreased from 91.4% pre-mepolizumab initiation to 88.6% post-mepolizumab initiation (*P* = .039). This effect was not observed after age stratification (Tables E1 and E2).

A significant reduction (28%) in the risk of medium-dose ICS use was observed pre- versus post-mepolizumab initiation, primarily driven by reductions observed in the adolescent cohort (Table I; see also Table E2). Although the risk of low-dose ICS use trended lower following mepolizumab initiation in the pediatric cohort (55% reduction) (Table E1), results were not significant.

**Fig 3:**
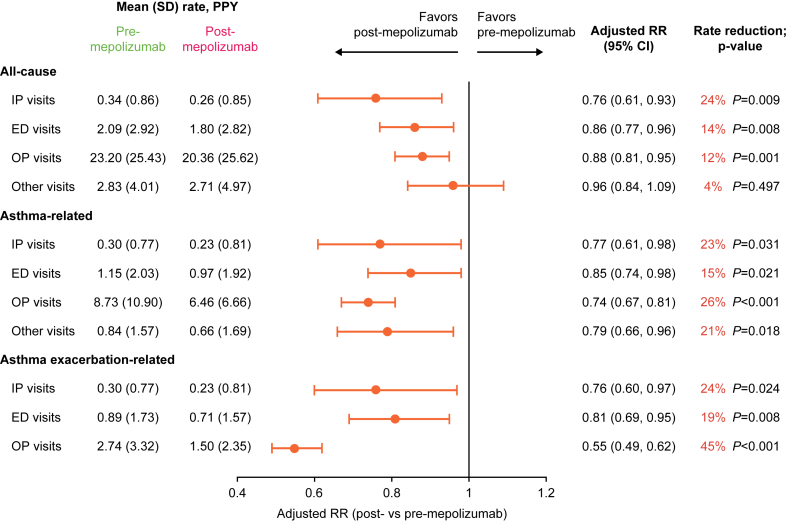
Rates of all-cause, asthma-related, and asthma exacerbation–related HCRU pre- and post-mepolizumab. *RR*, Rate ratio.

After initiating mepolizumab, the mean number of all-cause HCRU PPY significantly decreased by 24% for IP visits (*P* = .009), 14% for ED visits (*P* = .008), and 12% for OP visits (*P* = .001) (Fig 3). There was no significant change in all-cause other visits. Asthma-related HCRU PPY also showed significant reductions: 23% for IP visits (*P* = .031), 15% for ED visits (*P* = .021), and 26% for OP visits (*P* < .001) (Fig 3). In contrast to the all-cause category, the mean number of asthma-related other visits PPY significantly decreased by 21% (*P* = .018). A similar magnitude of HCRU reduction was observed for mean asthma exacerbation–related IP (24% reduction; *P* = .024) and ED (19% reduction; *P* = .008) visits PPY. The reduction in mean asthma exacerbation–related OP visits PPY (45% reduction; *P* < .001), however, was greater than that observed for the other 2 categories. When stratified by age, pediatric patients had significant decreases in the rates of all-cause, asthma-related, and asthma exacerbation–related OP visits but not other types of HCRU (Table E1); for adolescent patients, there were significant reductions in the rates of all types of visits except for all-cause OP and other visits (Table E2).

### Sensitivity analysis

The results among adolescent patients (12-17 years) for the period before the COVID-19 pandemic (n = 114) were overall consistent with the results during the entire study period (n = 305), except for HCRU, wherein the only significant changes prepandemic were reductions in the rates of asthma-related OP visits and asthma exacerbation–related ED and OP visits (Table E3).

## Discussion

This self-controlled cohort study is the first to use health care claims data to explore the real-world clinical and HCRU impact of mepolizumab initiation in a large, diverse, representative sample of children and adolescents with SA in the United States. Among these patients, more than 80% showed evidence of atopy and more than 25% had a food allergy; coexistence of asthma and food allergy has been identified as a risk factor for increased asthma-related HCRU and reduced lung function.^33^ A third of the patients included were Black and more than three-quarters were on Medicaid, which indicates that the study population is representative of the clinical need.^7^^,^^8^ Initiating mepolizumab in this patient population was associated with significant reductions in the rates of OCS dispensings (24%) and bursts (29%), asthma exacerbations (34%), and all-cause, asthma-related, and asthma exacerbation–related HCRU (up to 45%).

Exacerbations in SA are known to impose a substantial burden on the health care system,^34^ and children and adolescents with asthma have higher rates of IP hospitalizations and ED visits than adults.^35^ Real-world observational studies have reported that starting treatment with mepolizumab decreases HCRU in adult patients with SA, including primary care, specialist and ED visits, and IP hospitalizations, likely owing to associated reductions in the rate of exacerbations.^28^^,^^29^^,^^36^ Previous reports have indicated a positive impact of mepolizumab on HCRU in children, but data are limited.^37^^,^^38^ In a retrospective analysis of 16 patients (aged 7-17 years) comparing HCRU in the 12 months before and after starting mepolizumab, hospital admissions decreased by 67% (*P* = .007).^39^ Our results help fill the evidence gap regarding the impact of mepolizumab on exacerbations and HCRU in children and adolescents and how this treatment can alleviate the need for acute care. Such positive implications for reducing the economic burden associated with SA management have been previously shown in another US-based study reporting reductions in exacerbation-related hospitalizations following the initiation of mepolizumab and, subsequently, lower IP, OP, and ED costs.^25^

The effects of mepolizumab on asthma exacerbation rates and associated HCRU in our study are broadly consistent with those observed in clinical trials and other real-world studies.^23^, ^24^, ^25^, ^26^^,^^28^, ^29^, ^30^, ^31^ In a phase 2, open-label study in children aged 6-11 years with SA, mepolizumab decreased the annualized asthma exacerbation rate (defined by SCS use and/or IP hospitalizations and/or ED visits) to a mean of approximately 1.0 event per year^30^; the SCS-defined exacerbation rate was reduced to a mean of 1.3 events PPY in the post-mepolizumab period of the present study, and those defined by IP admission or ED visits decreased to a rate of 0.2 PPY. In the phase 2 MUPPITS-2 study of children and adolescents (6-17 years) with SA living in low-income urban areas, mepolizumab treatment decreased the annualized rate of SCS-treated asthma exacerbations by 27% compared with placebo (0.96 vs 1.30; rate ratio, 0.73 [95% CI, 0.56-0.96]; *P* = .027),^31^ an effect similar in magnitude to the 35% reduction observed in our study for SCS-defined exacerbations. Taken together, the evidence highlights the benefits of mepolizumab for alleviating the clinical burden associated with SA exacerbations in children and adolescents, as a therapy directly targeting underlying inflammation by inhibiting IL-5 and reducing the production and survival of eosinophils,^18^ and directly and indirectly affecting other inflammatory and structural cells within the airway.^21^ In addition, the findings from this study, in which 86% of children had comorbid allergic rhinitis, suggest that mepolizumab is effective in children with allergic asthma, which is the predominant asthma phenotype among children.^40^ The effect of mepolizumab on asthma exacerbations likely contributed to its positive impact on OCS treatment patterns; the clear link between improvements in the 2 outcomes has already been demonstrated in clinical trials and real-world studies of mepolizumab for SA.^22^, ^23^, ^24^, ^25^, ^26^, ^27^, ^28^, ^29^ Asthma treatment guidelines recommend administering short bursts of OCSs to manage SA exacerbations in adolescents and younger school-age children.^9^ OCSs are highly effective at rapidly reducing airway edema and secretions associated with acute asthma exacerbations^41^ and decrease hospitalization rates among children with SA.^42^, ^43^, ^44^ However, these benefits are offset by low tolerability and an undesirable side-effect profile, with vomiting, sleep disturbance, behavioral changes, adrenal suppression, and an increased risk of infections being common in children with even short courses of OCSs.^45^ As such, reducing OCS use remains a major goal of treatment when managing pediatric and adolescent patients with SA^9^ to improve quality of life while avoiding serious or irreversible complications.^24^^,^^45^

In the phase 3 Steroid Reduction with Mepolizumab Study (SIRIUS) trial, mepolizumab significantly reduced the median daily OCS dose by 50% compared with placebo (*P* = .007), accompanied by a 32% decline in the annual rate of exacerbations (*P* = .04)^24^; however, the lowest age of patients enrolled in the trial was 16 years. Another previous real-world, retrospective study in patients with allergic and nonallergic asthma found that mepolizumab initiation decreased OCS use, but data were not stratified by age and only a small number of patients were younger than 18 years.^23^ Thus, the results from our study provide important data supporting the steroid-sparing effect of mepolizumab (as shown by declining rates of OCS dispensings and bursts) in a large sample of children and adolescents (aged 6-17 years) with asthma. Although there was no significant effect of mepolizumab on maintenance or chronic OCS use, this may reflect the low number of patients using these treatment strategies long-term during the pre-mepolizumab period (11.0% and 7.4%, respectively), with subsequent impact on statistical power to detect a change.

The real-world effect of mepolizumab on ICS use has been investigated previously by Corren et al.^46^ They used administrative claims data to identify patients aged ≥12 years who had ≥2 mepolizumab administrations for asthma. The percentage of patients using ICSs decreased from 48% before initiating mepolizumab to 35% in the 12 months afterward. Our data show a decreasing trend in ICS dosage pre- to post-mepolizumab initiation, including a significant 28% reduction in the proportion of patients with ≥1 medium-dose ICS dispensings. Given the adverse effects associated with high-dose ICS use in children,^47^ additional real-world studies evaluating the potential ICS-sparing effect of mepolizumab in SA would be of value. In particular, it would be of use to determine whether children receiving medium-dose ICSs were able to reduce their dose to low-dose ICSs with mepolizumab treatment.

Regarding the use of rescue medications in our population, the numerically small but significant decline in the percentage of patients using ≥1 SABA canisters suggests decreased dependence on SABA following the initiation of mepolizumab. This is of interest given the negative association between excessive use of SABA and poor asthma outcomes (acute HCRU and mortality)^14^^,^^15^ and warrants exploration in future studies.

This study is strengthened by additional stratified analyses in which treatment with mepolizumab reduced the clinical and HCRU burden associated with SA irrespective of age subgroup (6-11 years and 12-17 years). Sensitivity analyses of adolescents in the period before the pandemic indicated no confounding effects of the COVID-19 pandemic (ie, the reduction of in-person services associated with stay-at-home mandates) on the study results and revealed similar trends to those found in the main study period. Although some HCRU outcomes did not show a statistically significant reduction post-mepolizumab, this is possibly due to the smaller sample size (n = 114); a downward trend following mepolizumab initiation was consistently observed for all HCRU outcomes.

This study had several limitations. First, the study was not designed to measure the clinical effectiveness of mepolizumab initiation precisely using functional indicators, such as peak expiratory flow or FEV_1_, because these data were not available in the administrative claims. Second, pharmacy claims only indicate that medications were dispensed but cannot confirm adherence or correct use by patients. Third, the correlation of within-patient observations during the pre- and post-mepolizumab periods was adjusted for using GEEs, and as a result, time-varying variables were not captured or controlled for within this analysis. In addition, single-inhaler maintenance and rescue therapy use was not captured in this study; given its increasing adoption in clinical practice, this may lead to an underestimation of rescue medications used. Finally, the study focused on pediatric and adolescent patients with SA with continuous health insurance enrollment for 2 years, reducing generalizability of the results. Notably, the study population was racially and ethnically diverse and most patients were covered by Medicaid, eligibility for which is predominantly determined by financial status, indicating the value of further exploration of social determinants of health related to asthma.

### Conclusion

This study provides comprehensive real-world evidence on the clinical and HCRU benefits of mepolizumab in a representative cohort of children and adolescents with SA, including reductions in OCS use, asthma exacerbations, SABA use, and HCRU. Future studies may provide additional insights by evaluating the comparative effectiveness of mepolizumab relative to other biologics for the treatment of SA and among pediatric and adolescent patients.

**Clinical implications**

Mepolizumab reduces exacerbations, OCS and SABA use, and HCRU in children/adolescents with SA, supporting its use in this population in real-world clinical practice.

## Disclosure statement

This study was funded by 10.13039/100004330GSK (GSK ID: 218952).

Disclosure of potential conflict of interest: M.L.H. has participated in an advisory board for GSK. T.C. and A.D. are employed by GSK and hold financial equities in GSK. F.L., M.M., A.H., and K.Z. are employees of Groupe d’analyse, Ltée, a consulting company that received payment from GSK to conduct this study.

Data-sharing statement: The data that support the findings of this study are available from Komodo Health, but restrictions apply to the availability of these data, which were subject to standard data licensing terms. The data are available through requests made directly to Komodo Health, subject to their respective requirements for access.
