# Supplementary material for: Real-world impact of mepolizumab on pediatric and adolescent patients with severe asthma
Source: J Allergy Clin Immunol Glob. 2025 Dec 20;5(2):100638. doi: 10.1016/j.jacig.2025.100638 (PMC12874570; doi:10.1016/j.jacig.2025.100638)
Supplement: Supplementary Data [file mmc1.docx]

**Supplementary Material**

### **Table E1. OCS treatment patterns, asthma exacerbations, SABA use, ICS dosage, and HCRU pre- versus post-mepolizumab: pediatric patients aged 6–11 years.**

|  | **Pre-mepolizumab period (N=275)** | **Post-mepolizumab period (N=275)** | **Measures of effect (95% CI)** | **P-value** |
| --- | --- | --- | --- | --- |
| **OCS treatment patterns** |  |  | **Rate ratio** |  |
| Number of OCS dispensings, PPY, mean ± SD [median] | 3.8 ± 3.4 [3.0] | 3.0 ± 3.6 [2.0] | 0.79 (0.71, 0.89) | <0.001 |
| Number of OCS bursts, PPY, mean ± SD [median] | 2.1 ± 1.9 [2.0] | 1.6 ± 1.7 [1.0] | 0.74 (0.66, 0.83) | <0.001 |
|  |  |  | **Risk ratio** |  |
| Patients with ≥1 OCS dispensing, n (%) | 241 (87.6) | 200 (72.7) | 0.83 (0.77, 0.89) | <0.001 |
| Maintenance OCS use, n (%) | 33 (12.0) | 28 (10.2) | 0.85 (0.59, 1.21) | 0.370 |
| Chronic OCS use, n (%) |  |  |  |  |
| Mean daily dose ≥5 mg | 16 (5.8) | 15 (5.5) | 0.94 (0.59, 1.48) | 0.782 |
| Mean daily dose ≥10 mg | 1 (0.4) | 2 (0.7) | 2.00 (0.50, 8.00) | 0.327 |
| Mean daily dose ≥10 mg during the last 90 days  of each period | 6 (2.2) | 7 (2.5) | 1.17 (0.43, 3.18) | 0.763 |
|  |  |  | **Mean difference** |  |
| Daily dose mg, mean ± SD [median] | 1.7 ± 1.7 [1.3] | 1.5 ± 1.9 [0.8] | −0.23 (−0.41, −0.05) | 0.012 |
| **Asthma exacerbations** |  |  |  |  |
| Rate of asthma exacerbations, PPY, mean ± SD [median] |  |  | **Rate ratio** |  |
| Overall exacerbation | 2.55 ± 2.14 [2.0] | 1.68 ± 2.04 [1.0] | 0.66 (0.59, 0.74) | <0.001 |
| SCS-defined exacerbation | 2.25 ± 1.99 [2.0] | 1.43 ± 1.73 [1.0] | 0.64 (0.56, 0.73) | <0.001 |
| IP/ED-defined exacerbation | 0.31 ± 0.76 [0.0] | 0.24 ± 0.82 [0.0] | 0.80 (0.57, 1.13) | 0.201 |
| **SABA use** |  |  | **Rate ratio** |  |
| Number of SABA canisters, PPY, mean ± SD [median] | 7.3 ± 5.6 [6.0] | 6.9 ± 6.3 [5.0] | 0.96 (0.88, 1.03) | 0.264 |
|  |  |  | **Risk ratio** |  |
| Patients with ≥1 SABA canister, n (%) | 260 (94.5) | 251 (91.3) | 0.97 (0.93, 1.00) | 0.083 |
| **ICS dosage, n (%)** |  |  | **Risk ratio** |  |
| Patients with ≥1 dispensing for low-dose ICS | 11 (4.0) | 5 (1.8) | 0.45 (0.17, 1.22) | 0.118 |
| Patients with ≥1 dispensing for medium-dose ICS | 42 (15.3) | 38 (13.8) | 0.90 (0.67, 1.21) | 0.505 |
| Patients with ≥1 dispensing for high-dose ICS | 249 (90.5) | 250 (90.9) | 1.00 (0.96, 1.05) | 0.847 |
| **All-cause, asthma-related and asthma exacerbation–related HCRU** |  |  |  |  |
| HCRU, PPY, mean ± SD [median] |  |  | **Rate ratio** |  |
| All-cause |  |  |  |  |
| IP visits | 0.35 ± 0.85 [0.0] | 0.29 ± 0.96 [0.0] | 0.83 (0.60, 1.15) | 0.270 |
| ED visits | 2.05 ± 2.75 [1.0] | 1.91 ± 3.09 [1.0] | 0.93 (0.80, 1.09) | 0.354 |
| OP visits | 24.05 ± 31.05 [16.0] | 20.69 ± 26.27 [13.0] | 0.86 (0.77, 0.96) | 0.005 |
| Other visits | 2.88 ± 3.66 [2.0] | 2.77 ± 4.68 [1.0] | 0.96 (0.81, 1.14) | 0.644 |
| Asthma-related |  |  |  |  |
| IP visits | 0.31 ± 0.78 [0.0] | 0.26 ± 0.90 [0.0] | 0.84 (0.58, 1.19) | 0.322 |
| ED visits | 1.08 ± 1.78 [0.0] | 1.01 ± 2.10 [0.0] | 0.94 (0.76, 1.16) | 0.581 |
| OP visits | 9.15 ± 13.08 [7.0] | 6.68 ± 6.59 [5.0] | 0.73 (0.62, 0.87) | <0.001 |
| Other visits | 0.91 ± 1.68 [0.0] | 0.80 ± 1.81 [0.0] | 0.88 (0.68, 1.12) | 0.292 |
| Asthma exacerbation–related |  |  |  |  |
| IP visits | 0.31 ± 0.79 [0.0] | 0.26 ± 0.90 [0.0] | 0.83 (0.58, 1.18) | 0.292 |
| ED visits | 0.87 ± 1.47 [0.0] | 0.73 ± 1.63 [0.0] | 0.85 (0.67, 1.07) | 0.169 |
| OP visits | 2.76 ± 2.77 [2.0] | 1.67 ± 2.53 [1.0] | 0.61 (0.51, 0.71) | <0.001 |

CI, confidence interval; ED, emergency department; HCRU, healthcare resource utilization; ICS, inhaled corticosteroid; IP, inpatient; OCS, oral corticosteroid; OP, outpatient; PPY, per patient-year; SABA, short-acting β_2_ agonist; SCS, systemic corticosteroid; SD, standard deviation.

### **Table E2. OCS treatment patterns, asthma exacerbations, SABA use, ICS dosage, and HCRU pre- versus post-mepolizumab: adolescent patients aged 12–17 years.**

|  | **Pre-mepolizumab period (N=305)** | **Post-mepolizumab period (N=305)** | **Measures of effect (95% CI)** | **P-value** |
| --- | --- | --- | --- | --- |
| **OCS treatment patterns** |  |  | **Rate ratio** |  |
| Number of OCS dispensings, PPY, mean ± SD [median] | 3.5 ± 3.7 [3.0] | 2.6 ± 3.8 [1.0] | 0.74 (0.64, 0.84) | <0.001 |
| Number of OCS bursts, PPY, mean ± SD [median] | 2.0 ± 1.9 [1.0] | 1.3 ± 1.7 [1.0] | 0.69 (0.61, 0.78) | <0.001 |
|  |  |  | **Risk ratio** |  |
| Patients with ≥1 OCS dispensing, n (%) | 238 (78.0) | 204 (66.9) | 0.86 (0.79, 0.93) | <0.001 |
| Maintenance OCS use, n (%) | 31 (10.2) | 26 (8.5) | 0.84 (0.59, 1.18) | 0.318 |
| Chronic OCS use, n (%) |  |  |  |  |
| Mean daily dose ≥5 mg | 27 (8.9) | 24 (7.9) | 0.89 (0.60, 1.31) | 0.549 |
| Mean daily dose ≥10 mg | 5 (1.6) | 3 (1.0) | 0.60 (0.17, 2.07) | 0.419 |
| Mean daily dose ≥10 mg during the last 90 days  of each period | 19 (6.2) | 8 (2.6) | 0.42 (0.21, 0.84) | 0.014 |
|  |  |  | **Mean difference** |  |
| Daily dose mg, mean ± SD [median] | 2.0 ± 2.3 [1.4] | 1.5 ± 2.2 [0.7] | −0.55 (−0.77, −0.33) | <0.001 |
| **Asthma exacerbations** |  |  |  |  |
| Rate of asthma exacerbations, PPY, mean ± SD [median] |  |  | **Rate ratio** |  |
| Overall exacerbation | 2.16 ± 2.08 [2.0] | 1.45 ± 1.93 [1.0] | 0.67 (0.59, 0.76) | <0.001 |
| SCS-defined exacerbation | 1.87 ± 1.83 [1.0] | 1.24 ± 1.65 [1.0] | 0.66 (0.58, 0.76) | <0.001 |
| IP/ED-defined exacerbation | 0.29 ± 0.75 [0.0] | 0.21 ± 0.72 [0.0] | 0.72 (0.54, 0.95) | 0.020 |
| **SABA use** |  |  | **Rate ratio** |  |
| Number of SABA canisters, PPY, mean ± SD [median] | 7.0 ± 6.0 [6.0] | 7.1 ± 6.4 [6.0] | 1.01 (0.93, 1.09) | 0.840 |
|  |  |  | **Risk ratio** |  |
| Patients with ≥1 SABA canister, n (%) | 270 (88.5) | 263 (86.2) | 0.97 (0.93, 1.02) | 0.223 |
| **ICS dosage, n (%)** |  |  | **Risk ratio** |  |
| Patients with ≥1 dispensing for low-dose ICS | 44 (14.4) | 41 (13.4) | 0.93 (0.69, 1.25) | 0.639 |
| Patients with ≥1 dispensing for medium-dose ICS | 135 (44.3) | 89 (29.2) | 0.66 (0.56, 0.78) | <0.001 |
| Patients with ≥1 dispensing for high-dose ICS | 240 (78.7) | 240 (78.7) | 1.00 (0.95, 1.06) | 1.000 |
| **All-cause, asthma-related and asthma exacerbation–related HCRU** |  |  |  |  |
| HCRU, PPY, mean ± SD [median] |  |  | **Rate ratio** |  |
| All-cause |  |  |  |  |
| IP visits | 0.34 ± 0.87 [0.0] | 0.23 ± 0.74 [0.0] | 0.69 (0.54, 0.88) | 0.003 |
| ED visits | 2.12 ± 3.07 [1.0] | 1.71 ± 2.56 [1.0] | 0.81 (0.69, 0.93) | 0.004 |
| OP visits | 22.43 ± 19.01 [16.0] | 20.05 ± 25.05 [14.0] | 0.89 (0.79, 1.00) | 0.060 |
| Other visits | 2.79 ± 4.30 [2.0] | 2.66 ± 5.23 [1.0] | 0.95 (0.79, 1.15) | 0.618 |
| Asthma-related |  |  |  |  |
| IP visits | 0.29 ± 0.75 [0.0] | 0.20 ± 0.71 [0.0] | 0.71 (0.53, 0.95) | 0.023 |
| ED visits | 1.21 ± 2.23 [1.0] | 0.94 ± 1.73 [0.0] | 0.78 (0.65, 0.93) | 0.005 |
| OP visits | 8.36 ± 8.47 [7.0] | 6.27 ± 6.72 [5.0] | 0.75 (0.69, 0.82) | <0.001 |
| Other visits | 0.77 ± 1.47 [0.0] | 0.54 ± 1.56 [0.0] | 0.71 (0.52, 0.96) | 0.025 |
| Asthma exacerbation–related |  |  |  |  |
| IP visits | 0.29 ± 0.75 [0.0] | 0.20 ± 0.71 [0.0] | 0.70 (0.52, 0.95) | 0.020 |
| ED visits | 0.90 ± 1.93 [0.0] | 0.70 ± 1.51 [0.0] | 0.77 (0.62, 0.95) | 0.017 |
| OP visits | 2.71 ± 3.75 [2.0] | 1.35 ± 2.16 [0.0] | 0.50 (0.42, 0.59) | <0.001 |

CI, confidence interval; ED, emergency department; HCRU, healthcare resource utilization; ICS, inhaled corticosteroid; IP, inpatient; OCS, oral corticosteroid; OP, outpatient; PPY, per patient-year; SABA, short-acting β_2_ agonist; SCS, systemic corticosteroid; SD, standard deviation.

### **Table E3. OCS treatment patterns, asthma exacerbations, SABA use, ICS dosage, and HCRU pre- versus post-mepolizumab for adolescent patients (aged 11–17 years) prior to the COVID-19 pandemic.**

|  | **Pre-mepolizumab period (N=114)** | **Post-mepolizumab period (N=114)** | **Measures of effect (95% CI)** | **P-value** |
| --- | --- | --- | --- | --- |
| **OCS treatment patterns** |  |  | **Rate ratio** |  |
| Number of OCS dispensings, PPY, mean ± SD [median] | 4.4 ± 4.1 [3.0] | 3.4 ± 4.8 [2.0] | 0.78 (0.64, 0.95) | 0.013 |
| Number of OCS bursts, PPY, mean ± SD [median] | 2.3 ± 2.0 [2.0] | 1.5 ± 1.8 [1.0] | 0.67 (0.56, 0.80) | <0.001 |
|  |  |  | **Risk ratio** |  |
| Patients with ≥1 OCS dispensing, n (%) | 98 (86.0) | 84 (73.7) | 0.86 (0.77, 0.96) | 0.006 |
| Maintenance OCS use, n (%) | 19 (16.7) | 16 (14.0) | 0.84 (0.60, 1.18) | 0.318 |
| Chronic OCS use, n (%) |  |  |  |  |
| Mean daily dose ≥5 mg | 17 (14.9) | 13 (11.4) | 0.76 (0.48, 1.21) | 0.250 |
| Mean daily dose ≥10 mg | 3 (2.6) | 3 (2.6) | 1.00 (0.27, 3.69) | 1.000 |
| Mean daily dose ≥10 mg during the last 90 days  of each period | 12 (10.5) | 7 (6.1) | 0.58 (0.27, 1.26) | 0.171 |
|  |  |  | **Mean difference** |  |
| Daily dose mg, mean ± SD [median] | 2.7 ± 2.6 [2.1] | 1.9 ± 2.8 [0.8] | −0.72 (−1.12, −0.32) | <0.001 |
| **SABA use** |  |  | **Rate ratio** |  |
| Number of SABA canisters, PPY, mean ± SD [median] | 7.9 ± 6.5 [7.0] | 7.1 ± 6.4 [6.0] | 0.89 (0.80, 1.01) | 0.062 |
|  |  |  | **Risk ratio** |  |
| Patients with ≥1 SABA canister, n (%) | 100 (87.7) | 96 (84.2) | 0.96 (0.90, 1.03) | 0.248 |
| **Asthma exacerbations** |  |  |  |  |
| Rate of asthma exacerbations, PPY, mean ± SD [median] |  |  | **Rate ratio** |  |
| Overall exacerbation | 2.61 ± 2.19 [2.0] | 1.89 ± 2.38 [1.0] | 0.72 (0.61, 0.86) | <0.001 |
| SCS-defined exacerbation | 2.19 ± 1.81 [2.0] | 1.55 ± 1.95 [1.0] | 0.71 (0.58, 0.86) | <0.001 |
| IP/ED-defined exacerbation | 0.41 ± 0.92 [0.0] | 0.33 ± 1.04 [0.0] | 0.81 (0.58, 1.12) | 0.198 |

| **All-cause, asthma-related and asthma exacerbation–related HCRU** |  |  |  |  |
| --- | --- | --- | --- | --- |
| HCRU, PPY, mean ± SD [median] |  |  | **Rate ratio** |  |
| All-cause |  |  |  |  |
| IP visits | 0.48 ± 1.14 [0.0] | 0.37 ± 1.05 [0.0] | 0.76 (0.56, 1.03) | 0.082 |
| ED visits | 2.25 ± 2.83 [1.0] | 1.82 ± 2.81 [1.0] | 0.81 (0.64, 1.03) | 0.085 |
| OP visits | 24.72 ± 20.10 [19.5] | 21.26 ± 24.75 [16.0] | 0.86 (0.72, 1.03) | 0.100 |
| Other visits | 3.20 ± 4.92 [2.0] | 3.01 ± 4.69 [1.0] | 0.94 (0.79, 1.12) | 0.496 |
| Asthma-related |  |  |  |  |
| IP visits | 0.42 ± 0.93 [0.0] | 0.32 ± 1.02 [0.0] | 0.77 (0.53, 1.11) | 0.165 |
| ED visits | 1.34 ± 2.37 [1.0] | 1.08 ± 1.98 [0.0] | 0.80 (0.63, 1.02) | 0.078 |
| OP visits | 9.66 ± 11.99 [8.0] | 7.64 ± 9.14 [6.0] | 0.79 (0.70, 0.90) | <0.001 |
| Other visits | 0.80 ± 1.23 [0.0] | 0.68 ± 1.70 [0.0] | 0.85 (0.54, 1.32) | 0.459 |
| Asthma exacerbation–related |  |  |  |  |
| IP visits | 0.42 ± 0.93 [0.0] | 0.32 ± 1.02 [0.0] | 0.77 (0.53, 1.11) | 0.165 |
| ED visits | 1.11 ± 2.31 [0.0] | 0.73 ± 1.64 [0.0] | 0.65 (0.48, 0.89) | 0.006 |
| OP visits | 3.54 ± 4.62 [2.0] | 1.85 ± 2.51 [1.0] | 0.52 (0.41, 0.67) | <0.001 |

CI, confidence interval; ED, emergency department; HCRU, healthcare resource utilization; ICS, inhaled corticosteroid; IP, inpatient; OCS, oral corticosteroid; OP, outpatient; PPY, per patient-year; SABA, short-acting β_2_ agonist; SCS, systemic corticosteroid; SD, standard deviation.

**Figure E1. Study design.**


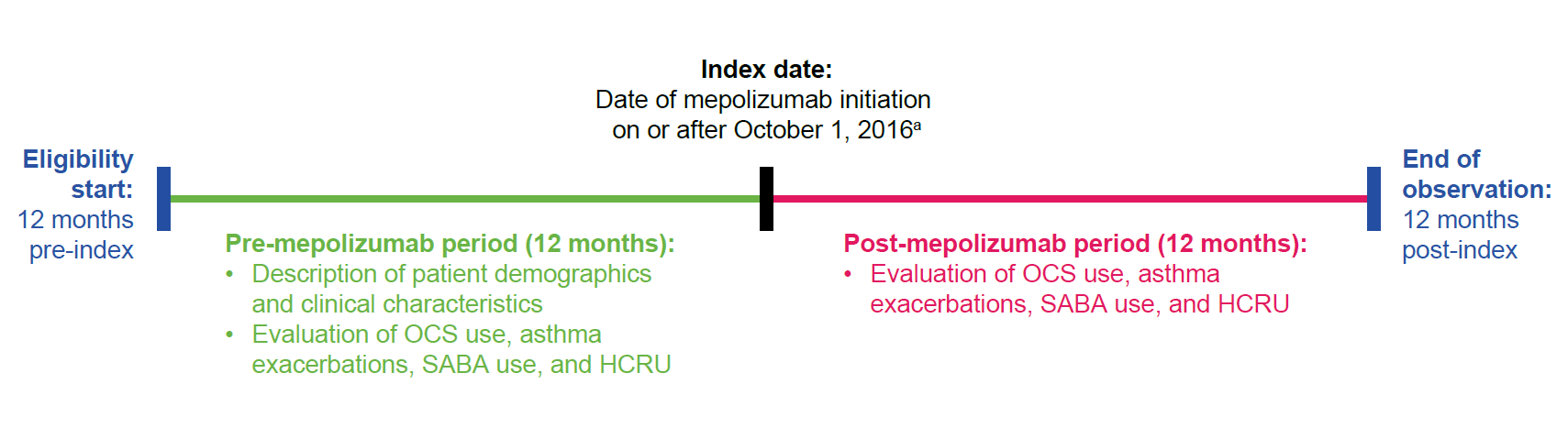


HCRU, healthcare resource utilization; OCS, oral corticosteroid; SABA, short-acting β_2_ agonist.

^a^Mepolizumab initiation was identified on or after October 1, 2016, to allow for a 12-month period prior to the index date.
